# Federated learning for critical electrical infrastructure—handling data heterogeneity for predictive maintenance of substation equipment

**DOI:** 10.3389/frai.2025.1697175

**Published:** 2026-01-27

**Authors:** Soham Ghosh, Gaurav Mittal

**Affiliations:** 1 Department of Electrical Engineering, Black and Veatch, Overland Park, KS, United States; 2 Department of Enterprise Solutions, Black and Veatch, Overland Park, KS, United States

**Keywords:** federated learning, preventive maintenance, substation maintenance, distributed learning, federated information criterion

## Abstract

High-voltage substations form the backbone of critical electrical infrastructure, making predictive maintenance essential for ensuring grid resilience and operational reliability. Federated learning (FL) presents an innovative strategy for predictive maintenance, allowing multiple utility providers to improve model performance jointly while maintaining data confidentiality. Rather than transmitting raw records, each electrical utility performs local model updates and shares only the refined parameters, thereby safeguarding sensitive information and capitalizing on the heterogeneity of equipment conditions across sites. This study develops a set of privacy-preserving FL frameworks to enhance preventive maintenance of substation circuit breakers, large power transformers, and emergency generators. It rigorously tackles the issue of data heterogeneity arising from variations in distribution patterns across utilities, an inherent challenge that hampers effective collaborative model development. Four FL strategies—Federated Averaging (FedAvg and FedAvgM), Federated Proximal (FedProx), and Federated Batch Normalization (FedBN), are evaluated for robustness against distributional shifts. Model performance in this study is evaluated using the F-score, which for the non-IID case ranges from 0.60 to 0.88 depending on the number of clients, the federated learning algorithm used, and the non-IID partitioning strategy employed. Also, a first-of-a kind Federated Information Criterion (FIC) is proposed in this manuscript as an extension of the classical information criterion. The results demonstrate that FedBN is best suited in mitigating cross-utility heterogeneity, yielding highest F-score of 0.88 and a moderately low FIC score of 4.35. Such tailored FL methods significantly improve predictive accuracy, enabling scalable and privacy-preserving deployment of FL in critical power system applications.

## 1 Introduction

Federated Learning (FL) is gaining traction as a valuable framework for predictive maintenance (PdM), especially in industrial applications, by allowing decentralized stakeholders to build shared machine learning models without disclosing proprietary or sensitive data. Though current wide scale adoption is limited and most of the research literature heavily focuses on performance benchmarking (du Ogier Terrail and Samy-Safwan, 2022; Ye et al., 2024) on standard datasets such as CIFAR-10, MNIST, Clinic10, the federated learning methodology has tremendous potential and is especially suitable for industries (Boobalan et al., 2022; Ramírez et al., 2023) characterized by distributed data sources that face legal and operational constraints regarding data sharing. The capabilities of FL align with the needs of predictive maintenance, where timely and effective analysis of operational data can significantly reduce downtime and maintenance costs.

A key advantage of using federated learning in predictive maintenance lies in its decentralized nature, which enables insights to be drawn from distributed datasets without requiring aggregation at a central repository. For instance, Bharti and McGibney (2021) emphasized that FL enables collaborative model development across independent organizations while ensuring that proprietary information remains local, thus mitigating common privacy and security risks in industrial environments. This feature became particularly relevant with the advent of Industry 4.0, where manufacturing and operational assets generate vast amounts of data stored across various silos (Bemani and Björsell, 2023).

In addition, several novel frameworks have been introduced to tackle issues related to inconsistent data distributions and limited accessibility commonly encountered in predictive maintenance. For example, Wahl et al. (2024), introduces an asynchronous federated learning approach that is sensitive to data disparity and temporal unavailability of training sets, which is critical for transportation fleet maintenance. The techniques they introduced seek to optimize the learning process, improving forecast precision and supporting proactive maintenance planning. These developments underscore how federated learning can be tailored to meet practical demands in equipment monitoring and failure prevention. The work of Li et al. (2020), further underscores this, discussing various strategies for federated optimization under heterogeneous data conditions and highlighting that FL’s flexibility can be transformative in environments where data characteristics significantly differ from one device or organization to another. From a field deployability standpoint, the integration of edge computing with FL models facilitates more rapid and accurate maintenance decisions, aligning with the operational needs for competency and efficiency in industrial settings (Sun et al., 2021).

Recent empirical studies have supported the feasibility and effectiveness of these FL applications. For example, Ahn et al. (2023), reported strong predictive performance in maintenance applications by integrating decentralized model training with temporal anomaly identification methods, suggesting significant potential for deployment in practical settings. The adaptive application of FL in PdM contexts not only showcases its potential for enhancing predictive modeling but also its critical role in supporting organizations in achieving operational excellence. As such, federated learning is not merely a privacy-preserving alternative, but a fundamentally more viable and operationally aligned approach for predictive maintenance in power systems. Table 1 summarizes a clear distinction between traditional centralized learning and federated learning (FL) in the context of predictive maintenance for power system apparatus.

**Table 1:** Centralized learning vs. federated learning for predictive maintenance.

Aspect	Centralized learning	Federated learning
Data aggregation	If deployed, raw sensor data from all clients (utilities) is transmitted to a central server.	Data remains local; only model updates or gradients are shared.
Data privacy	High risk due to transfer of sensitive operational data across utility boundaries.	Preserves data privacy; raw measurements never leave the local utility.
Regulatory and compliance risks	If deployed, may violate utility-specific data governance or interconnection policies.	Aligns with strict data protection protocols and utility-specific constraints.
Scalability	The learning process may become capped by data transfer bandwidth and centralized storage/processing limitations.	Federated learning is naturally scalable; computation is distributed across participating clients.
Robustness to data heterogeneity	Typically assumes IID data; performance may degrade under client-specific non-IID conditions.	Federated learning models are designed to handle non-IID settings; algorithms like FedProx and FedBN explicitly mitigate heterogeneity.
Model performance adaptability	Single model may underperform on minority or skewed utility-specific distributions.	FL allows personalized or clustered models better suited to each utility’s local data distribution.

While recent experimental research has highlighted both the practicality and impact of decentralized learning approaches such as Federated Learning (FL) across various domains, its widespread adoption still remains somewhat limited. FL has primarily gained traction in sectors such as healthcare (Chaddad et al., 2024; Rieke et al., 2020) and finance (Shi et al., 2023), with emerging use cases in predictive maintenance within the automotive and manufacturing industries (da Silveira Dib et al., 2021). In the field of electrical engineering, FL applications have remained confined at a theoretical level or have witnessed limited small scale residential or community level implementations in the areas of

- non-intrusive load monitoring (Wang et al., 2021; Giuseppi et al., 2022),
- energy theft detection (Wen et al., 2022; Ashraf et al., 2022),
- residential level demand forecasting (Zhao et al., 2021; Dasari et al., 2021; He et al., 2021), and.
- voltage control through reactive power injection (Zhao et al., 2023).

However, large-scale FL implementation, particularly in power delivery predictive maintenance applications at transmission and distribution substation level, remain sparse. Electrical substations form the backbone of the power grid and the gap highlights vast adoption potential of these FL frameworks in the domain of substation predictive maintenance. A survey conducted by the authors revealed that only 2 of the 24 major U.S. electric utilities have previously piloted a federated learning–based preventive maintenance program. This limited uptake is partially attributable to the traditionally siloed operational and maintenance structures of electrical utilities, both in the United States and internationally, as well as the steep technological learning curves associated with deploying FL frameworks in legacy infrastructure. These challenges form the motivation for the present study.

(a) Motivation towards application of FL in utility level power system applications

Federated Learning (FL) presents a transformative opportunity for power system applications by enabling privacy-preserving, distributed model training across utilities and substations, an approach that directly addresses the limitations of traditional predictive maintenance and asset management strategies. The motivation for adopting FL in this domain stems from the following key factors:

1. Outdated and labor-intensive maintenance practices: Many utilities continue to rely on manual inspections, portable dissolved gas in oil analysis, vibration analysis, infrared thermography, or static metadata (e.g., equipment nameplate information) (Cazacu et al., 2018; Mobley, 2002; Nazmul Huda and Taib, 2013; Molęda et al., 2023) for asset assessment—methods that are reactive, infrequent, and not scalable for aging infrastructure.
2. Limitations of centralized AI models: Existing AI-driven tools have rapidly evolved in the last several years and often use a blend of algorithms for predictive maintenance functions (Ghosh and Dutta, 2021; Hung, 2021; Beretta et al., 2021; Swier et al., 2025). However, these algorithms are typically trained in a central static environment, offering limited adaptability to localized asset behavior or environmental variation. They lack the continuous learning capability that federated frameworks inherently support.
3. Barriers to data sharing across utilities: Due to siloed operations, regulatory restrictions, and cybersecurity concerns, utilities are often unable to share raw data (Lee et al., 2019). FL supports collective model development across dispersed stakeholders while keeping sensitive information localized, offering a viable approach aligned with the inherently distributed nature of the energy industry.

(b) Manuscript contributions

To the best of our knowledge, no prior studies have extensively explored the application of federated learning for predictive maintenance in power systems engineering, and especially in the niche area of high voltage substations at the individual equipment level, while simultaneously addressing the practical challenges posed by data heterogeneity across decentralized sensor networks. This study offers several novel insights that strengthen the use of decentralized learning techniques for predictive maintenance within the power infrastructure domain, specifically addressing key challenges associated with data heterogeneity, infrastructure monitoring, and decentralized model training. The key advancements presented in this manuscript are:

1. The analysis is grounded in sensor-level data collected from critical substation equipment, including high-voltage circuit breakers, large power transformers, and emergency generators, offering a realistic and operationally relevant foundation for modeling.
2. The study explicitly characterizes data heterogeneity through both label skew (variation in failure class distributions across clients) and feature skew (differences in sensor measurement spaces), providing a nuanced understanding of real-world non-IID conditions.
3. The study evaluates a suite of federated learning algorithms designed to handle heterogeneity, such as FedProx, FedBN, and FedAvgM, conducting a comparative performance analysis to assess their robustness across diverse client conditions.
4. The study offers practical recommendations on mitigating heterogeneity during the experimental setup phase, including client clustering and sensor harmonization strategies.
5. The study introduces a novel Federated Information Criterion (FIC), the first of its kind to the best of our knowledge, which extends classical model selection frameworks by incorporating not just model fit and complexity, but also communication cost and heterogeneity penalties.

Collectively, these contributions establish a rigorous, scalable, and domain-specific foundation for implementing FL in electric utility maintenance workflows.

**Figure 1:**
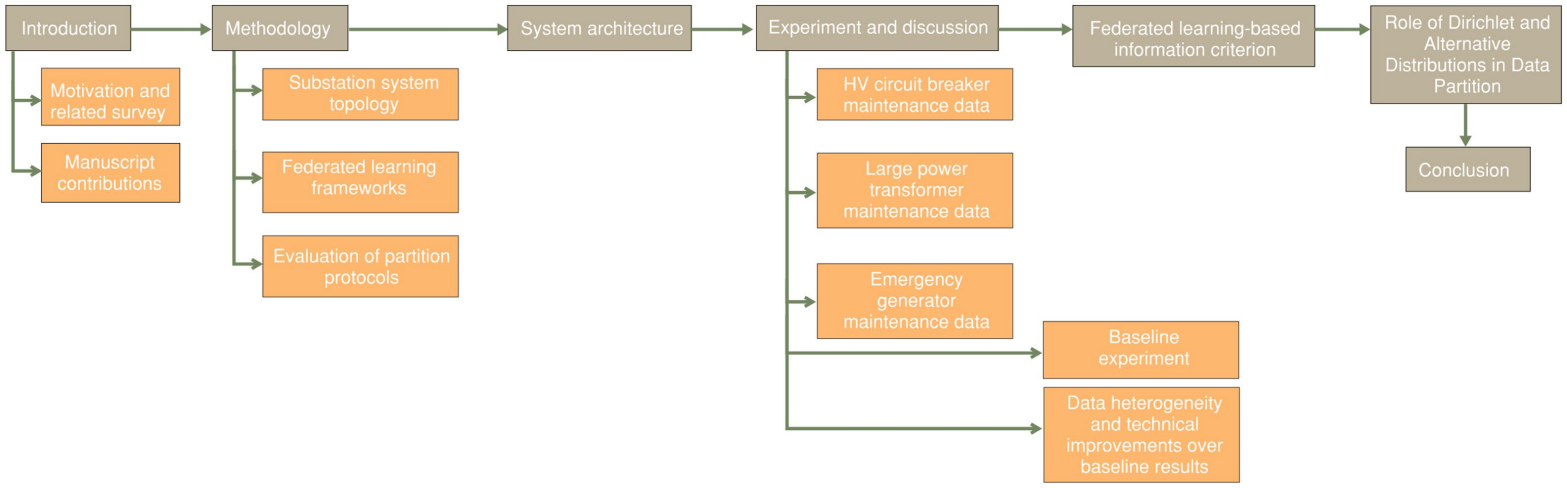
Manuscript’s structural organization.

The remainder of this manuscript is organized as follows, with a visual outline provided in Figure 1. Section II outlines the overall methodology and system architecture relevant to electrical substations, detailing the data acquisition process from critical assets such as circuit breakers, transformers, and emergency generators. It further examines leading decentralized learning approaches, offering a comparative analysis of their architectural adaptations to handle data heterogeneity across clients. Section III goes a little more into the details of the system architecture and in path for the flow of information between multiple utility substations and centralized training servers. Section IV describes the three domain-specific datasets used in this study and presents the baseline experimental setup using FedAvg and FedAvgM under data heterogeneity conditions. This section also examines how sophisticated federated learning techniques, FedBN and FedProx, perform under varying conditions and data distributions. These algorithms are tailored for heterogeneous settings, and their model performance are compared based on F1 scores and empirical observations. Inference-driven recommendations for improving FL deployment in operational utility contexts are also provided. Section V introduces the proposed Federated Information Criterion (FIC), a novel model selection metric that jointly considers model fit, complexity, communication cost, and data heterogeneity. Section VI presents a discussion on Dirichlet and alternative distributions as it applies to federated learning and related data partitions. Finally, Section VII concludes the paper by summarizing key findings and outlining directions for future research, including extensions toward personalized federated learning and integration into broader utility asset management systems.

## 2 Methodology

### 2.1 Outline of a substation system topology

High voltage circuit breakers, large power transformers, and emergency substation generators are critical components (Krieg, 2019) of high voltage power delivery systems, ensuring the safe, reliable, and continuous transmission of electricity across vast networks. Circuit breakers protect the grid by isolating faults and preventing equipment damage, while power transformers enable efficient voltage regulation for long-distance transmission and distribution. Station emergency generators provide essential backup power to maintain operational stability during outages and extend the duration of scheduled maintenance if backup stations service feed is unavailable. Together, these assets form the backbone of resilient and secure electric power infrastructure. Hence it is imperative that the electrical utilities collaborate in some fashion to enhance the predictive maintenance of these critical grid infrastructures through collaborative federated learning techniques.

At a component level, predictive maintenance of substation circuit breakers is critical to ensuring the stability of modern power systems. As a use case, in a distribution substation supporting hyperscale data center cluster, continuous monitoring and proactive maintenance of 138 kV line-tie breakers and 34.5 kV feeder breakers are essential. Failure of any of these breakers to operate correctly during fault conditions may result in disturbances persisting long enough for the data center to disconnect from the grid and transition to on-site uninterruptible power supplies (UPS). The disconnection of large data center loads, typically ranging from 500 MW to 1.5 GW, can trigger significant over-frequency events that threaten overall grid stability. Federated learning-based predictive maintenance frameworks enable diverse utilities to collaboratively improve predictive models without compromising data privacy, offering an automated, scalable, and privacy-preserving solution for enhancing breaker reliability in this critical context.

**Figure 2:**
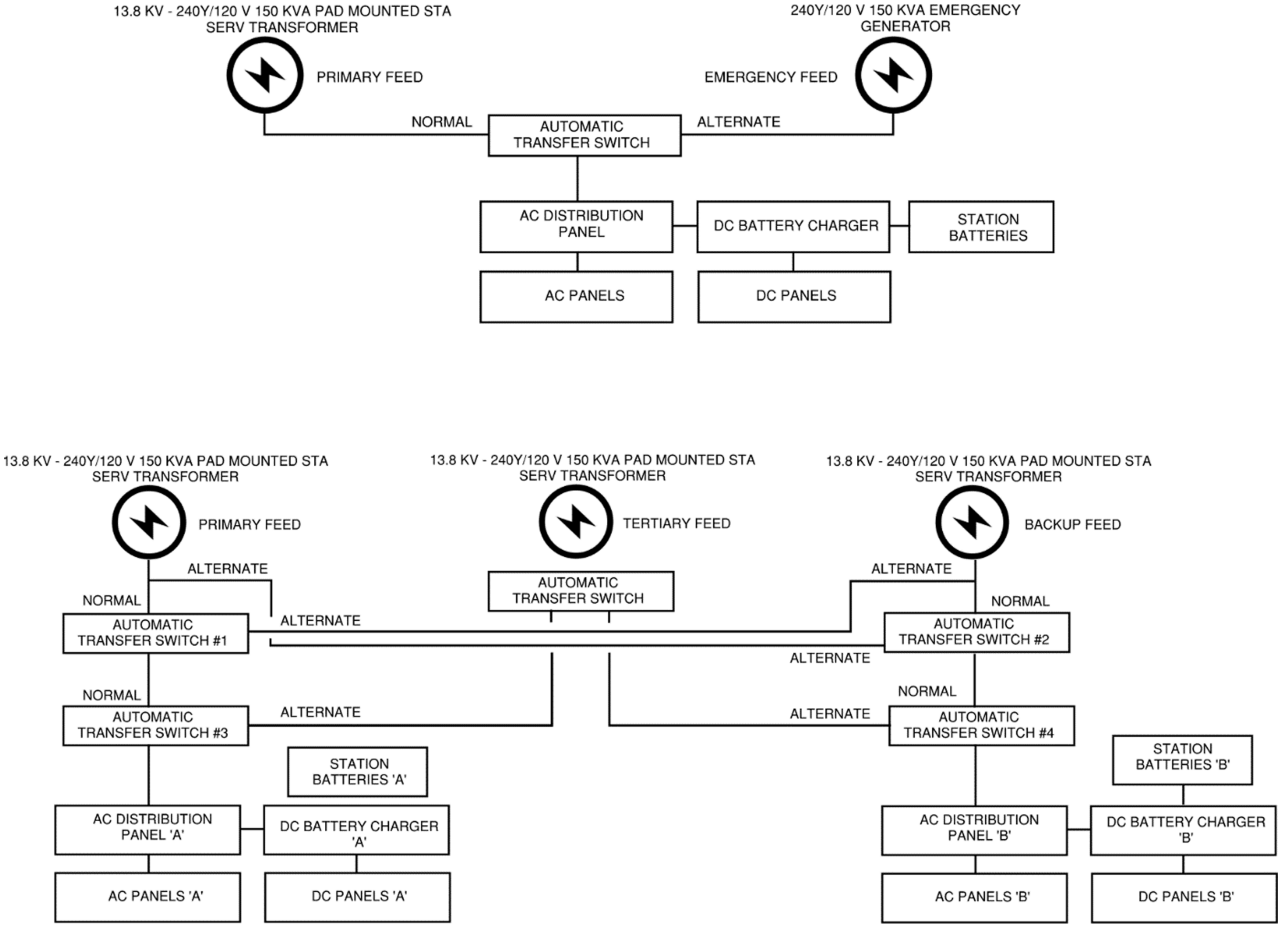
Station service single line with utility and emergency generator feeds. (top) emergency generator serving as the alternate feed given the unavailability of a local utility, (bottom) emergency generator serving as a tertiary feed given both the station services are from the same source (substation).

The second focus of analysis is on high-capacity transformers, which rank among the most vital and high-cost elements within a substation’s infrastructure, with replacement costs typically ranging from $10 to $15 million and procurement lead times of 115 to 130 weeks (Nguyen et al., 2022; Metwally, 2011). Catastrophic failure of a transformer not only imposes severe financial penalties but also jeopardizes system reliability over extended durations. Given their strategic role and the high cost of downtime, predictive maintenance enabled by federated learning provides a compelling solution. The third item, substation emergency generators, also falls under the critical asset category, as their timely operation ensures that station protection, control, and communication systems are preserved during outages or maintenance operations. Emergency generators in the context of substation are commonly used in two scenarios, a. when there is no backup station service source from a local utility feed given the remoteness of the site and constructing one is prohibitively expensive, and b. when both the primary and backup stations service feeds are from the substation itself, a tertiary power source from an emergency generator might be needed for redundancy purposes, with Figure 2 showing the single line network of both these topologies.

**Figure 3:**
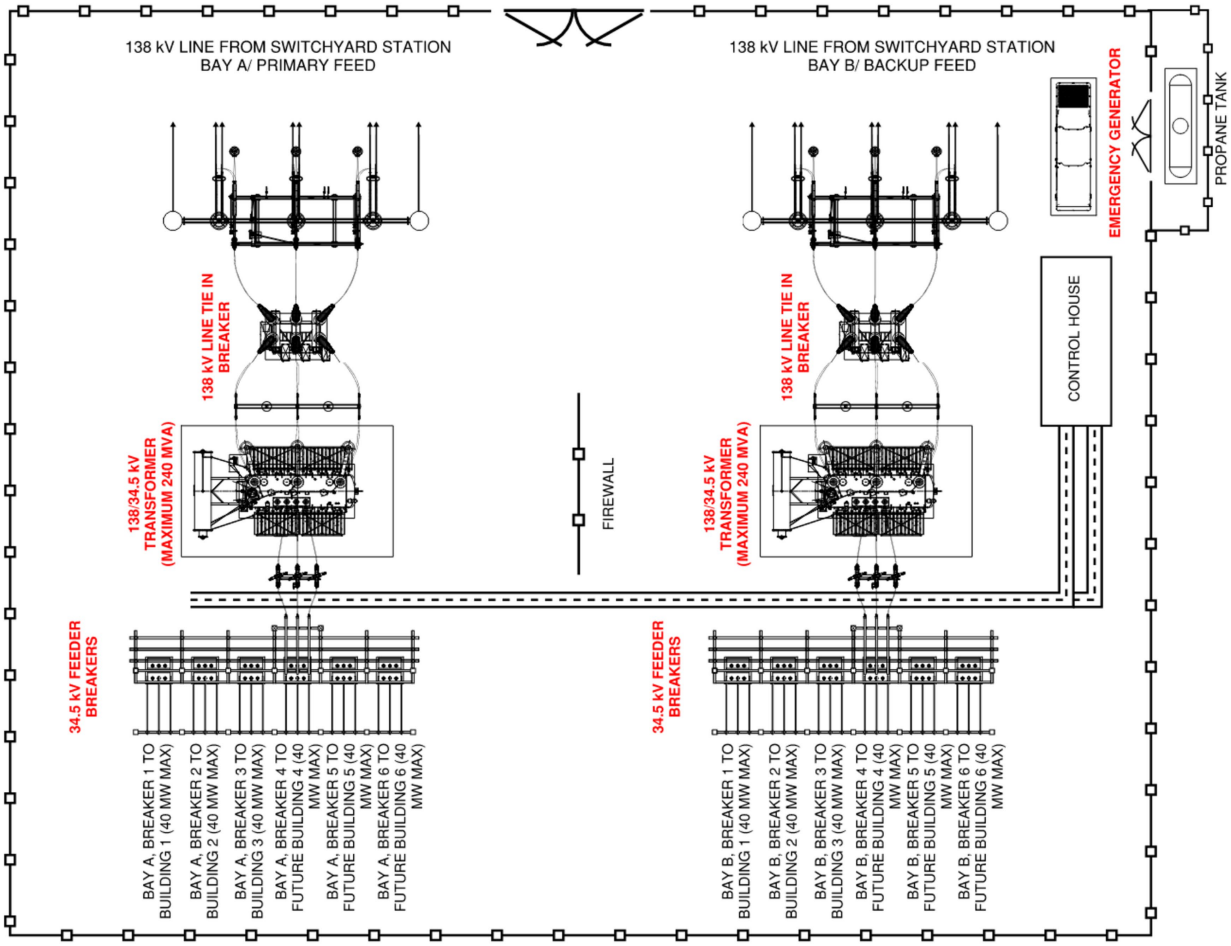
A 138/34.5 kV distribution substation for a mission critical data center. In red ink, the 138 kV main breakers and 34.5 kV feeder breakers, the 138/34.5 kV power transformer, and the emergency generator are deemed to be critical station assets.

Figure 3 shows a test bed 138/34.5 kV substation arrangement serving a data center facility, with main line tie-in breakers at 138 kV, large 180/220/240 MVA power transformers, and 34.5 kV feeder breakers. Each of the feeder breakers supply a data center building; with the data center’s ability to handle electronic faults usually being governed by the ITIC or CBEMA curves (Honrubia-Escribano et al., 2012; Gomex and Morcos, 2002; Heydt, 1998). Implementing predictive maintenance can help ensure that the feeder breakers and main line tie-in breakers trip as intended and driven by the substation protections scheme within three to five cycles, thereby enabling the data center to withstand disturbances during fault events.

### 2.2 FL implementation framework and evaluation of partition protocols

Now that the system topography of such electrical substations is understood, the focus shall be shifted to implementation framework. Federated learning (FL) has seen rapid advancements in implementation frameworks, lowering the barrier for deploying privacy-preserving machine learning systems. TensorFlow Federated (TFF), developed by Google, offers one of the most seamless integrations into existing machine learning workflows by extending the widely used TensorFlow and Keras ecosystems. TFF abstracts the complexities of distributed optimization, allowing users to define models using familiar Keras APIs and then apply federated computations with minimal modification. Its modular design supports both simulated federated learning (on centralized data partitioned to mimic clients) and deployment to real-world distributed systems. Built-in support for non-IID partitioning, custom aggregation strategies, and differential privacy integration further enhances its flexibility. Unlike several other frameworks that either require learning new syntaxes or lack deep backend integration with production ML tools, TFF maintains native interoperability with TensorFlow Serving, TFRecords, and Keras model export, making it exceptionally versatile for both research prototyping and scalable production deployment.

Though this study employs TensorFlow Federated for model development and experimentation, the authors have conducted an in-depth review of alternative federated learning frameworks and documented their core features and limitations in Tables 2, 3 for comparative context.

**Table 2:** Evaluation of federated learning frameworks in terms of workflow integration, system scalability, and privacy-preserving capabilities (Ziller et al., 2021; Saidani, 2023; Riedel et al., 2024).

Framework	Language(s)	Core strengths	Ease of integration	Key features	Limitations
TensorFlow Federated (TFF)	Python (TensorFlow)	Seamless Keras/TensorFlow integration	Very high	Native Keras support, differential privacy, custom aggregators, simulation + real-world deployment	Requires TensorFlow environment; limited direct mobile deployment support
FedML (TensorOpera AI)	Python	Flexible hardware support (edge, cloud), cross-platform	Moderate	Cross-device FL, cross-silo FL, benchmarking tools	More complex API surface; steeper learning curve
Flower	Python, (partial support for Java, Go)	Lightweight, highly customizable	High	Device heterogeneity handling, client sampling, minimal setup	Requires manual model wrapping, basic ML library abstraction.
PySyft	Python (PyTorch primarily)	Privacy-preserving computation (secure multi-party computation, differential privacy)	Moderate	Encrypted computation, data privacy first, multi-backend support	Heavy emphasis on privacy may complicate general FL tasks. Documentation and features are not well maintained.
OpenFL	Python	Enterprise-grade FL for healthcare and manufacturing	Moderate	Security-first, Intel optimizations, Docker-based deployment	Less flexible for non-enterprise use cases; steeper setup
Flute	C++/Python	High scalability, production-grade FL	Low	Production at hyperscale (Azure), asynchronous updates	Limited documentation; specialized for Microsoft Azure ecosystems

**Table 3:** Availability of partitioning protocol and non-IID algorithms between different federated learning frameworks. 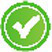 available, 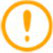 beta or not fully supported, 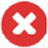 unknown or sufficient documentation not available.

Attributes	TFF	FedML	Flower	PySyft	OpenFL	Flute
Skewing type						
Label skew	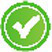	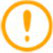	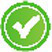	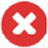	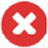	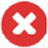
Feature skew	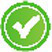	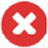	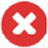	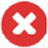	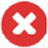	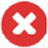
Quantity skew	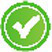	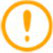	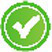	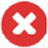	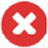	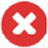
Data heterogeneity solutions						
FedAvgM	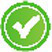	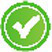	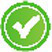	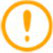	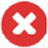	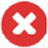
FedProx	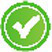	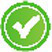	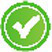	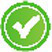	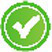	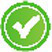
FedBN	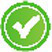	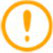	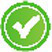	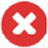	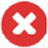	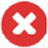
SCAFFOLD	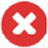	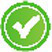	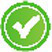	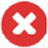	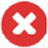	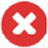
FedDyn	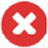	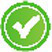	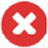	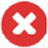	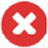	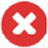

At the time of writing this manuscript, all the above frameworks were actively maintained. User should always check the developer-controlled version for availability and support of these features and functionality.

## 3 System architecture

**Figure 4:**
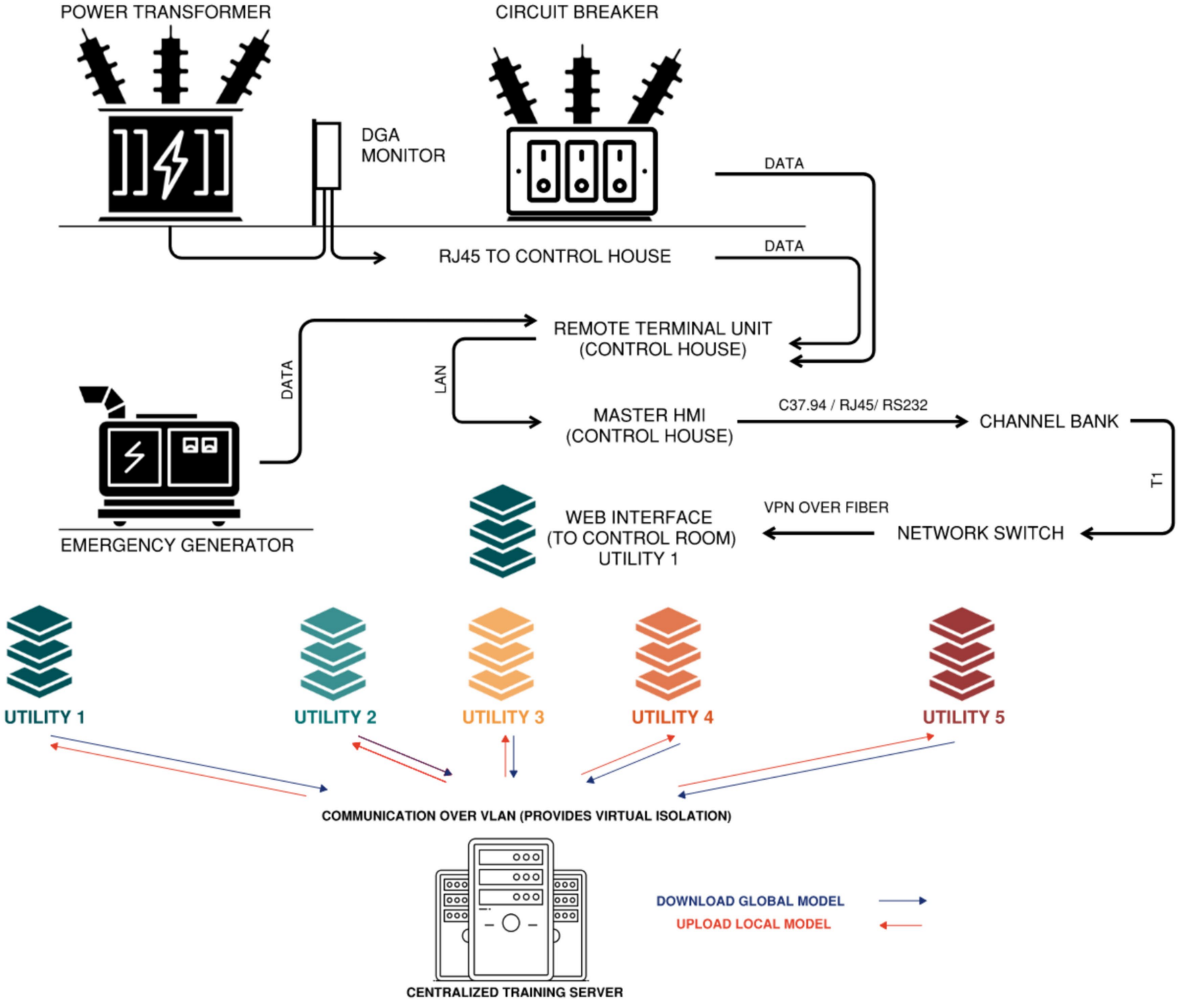
Telecommunications path (configuration may vary on use case) from substation equipment to utility control room via remote terminal unit and master HMI. Participating utilities collaborate with centralized training server over virtual LAN network.

In this study, we apply federated learning to sensor measurement data collected from high-voltage circuit breakers, large power transformers and their associated dissolved gas monitors, and emergency generators. These sensor measurements are initially collected by a remote terminal unit (RTU) (Madonsela et al., 2018) located in the substation control house. The RTUs aggregate and organize the data, which is then transmitted to a channel bank for further processing. From the channel bank, the date is packaged and forwarded through a network switch, ultimately reaching the utility’s centralized control room for operational monitoring; see Figure 4.

In a federated learning setup, an aggregated model is maintained by a coordinating node that orchestrates updates from distributed participants. The aggregated model evolves through contributions from individually trained models developed by each participating utility. These entities perform local training using proprietary sensor measurements, ensuring that no unprocessed data leaves their premises. Rather than transmitting raw inputs, they share parameter updates, thereby maintaining data confidentiality. The global model learns from the collective knowledge, the “wisdom of the crowd,” and iteratively refines itself based on diverse asset behaviors and conditions across the participating utilities. The updated and increasingly accurate predictive models are then disseminated back to the utilities’ control room, enhancing the predictive maintenance and operational reliability of critical substation assets.

## 4 Experiments and discussion

(a) Dataset description

For the purpose of this study, datasets were generated from sensor data to emulate a cluster of diverse electrical utilities and were tailored to form three databases, as described in the following paragraphs:

i HV Circuit Breaker Maintenance Data

1. The dataset is derived by combining results from high-voltage breaker monitoring (115–345 kV, with some 34.5 kV assets), where sensors capture critical measurements such as SF6 density, breaker status, and ambient cabinet temperature.
2. Engineers at participating anonymized virtual utilities routinely reviewed breaker records and associated measurements for assets under their operational oversight and flagged cases requiring maintenance. These inspection-driven flags are typically based on factors such as SF6 dew point, SF6 density, fault operation count, clearing time, and days since last operation, in accordance with each utility’s established maintenance standards.
3. The dataset comprises 5,000 samples of breaker readings, aggregated from five representative utilities. Each breaker is assigned a unique categorical identifier corresponding to its source utility, with 28 features representing various sensor-based measurements and one quality metric (0: no maintenance required, 1: maintenance required).
4. It is important to note that not all 28 features are uniformly available across all product variants, leading to slight variations in the feature space between variants.
5. To enhance the training of machine learning models, the dataset has been augmented with an increased proportion of ‘maintenance required’ examples to ensure sufficient representation of predictive maintenance cases.

ii Large Power Transformer Maintenance Data

1. The dataset is similar to the *HV Circuit Breaker Maintenance Data Set* and is derived by combining results from large power transformer monitoring, where sensors capture critical measurements such as LTC and main tank oil temperature, dissolved gas values (in ppm).
2. Engineers at participating utilities routinely reviewed transformer records and associated measurements for assets under their operational oversight and flagged cases requiring maintenance. These inspection-driven flags are typically based on factors such as high oil temperature, and excessive amount of certain dissolved gas (usually based on Duval triangles and pentagons (Akbari et al., 2008; Cheim et al., 2020)), following each utility’s established maintenance standards.
3. The dataset comprises 7,500 samples of breaker readings, aggregated from five representative utilities. Each transformer is assigned a unique categorical identifier corresponding to its source utility, with 18 features representing various sensor-based measurements and one quality metric (0: no maintenance required, 1: maintenance required).

iii Emergency Station Generator Maintenance Data

1. The dataset is derived by combining results from station emergency generators (~150–300 kVA on propane, natural gas, or diesel), where sensors capture critical measurements such as engine temperatures, oil pressure, alternate current and voltages.
2. Engineers at participating utilities routinely reviewed station emergency generator records and associated measurements for assets under their operational oversight and flagged cases requiring maintenance. These inspection-driven flags are typically based on factors such as start attempts, battery state of charge, lube oil temperature, crankcase pressure, and emission levels, in accordance with each utility’s established maintenance standards.
3. The dataset comprises 1,200 samples of emergency generator readings, aggregated from four representative utilities. There are 32 features representing various sensor-based measurements and one quality metric (0: no maintenance required, 1: maintenance required).

(b) Baseline experiment and discussion

To understand the rationale for introducing federated learning in predictive maintenance, we evaluated a centralized logistic regression model using the *HV Circuit Breaker Maintenance Dataset* under IID conditions. We randomly selected 1,000 IID samples for training and 500 IID samples for testing, ensuring no overlap between the sets. The centralized IID logistic model achieved an accuracy of 0.80 on the IID test set using all covariates from the data set.

To simulate heterogeneity under non-IID configuration, we sampled data from five utilities with varying equipment and operating conditions using the same *HV Circuit Breaker Maintenance Dataset.* We first tested the centralized model trained on IID data on this non-IID dataset. The accuracy of the IID model dropped to 0.77, highlighting the impact of non-IID assumption violations of a logistic regression model. To account for utility-level variance, we trained a logistic regression model with random effects on utility-level clustering. This approach improved accuracy slightly to 0.78. While stratified modeling helped marginally, it was insufficient to fully address the challenges of non-IID data. These results emphasize the need for more robust approaches like federated learning.

**Figure 5:**
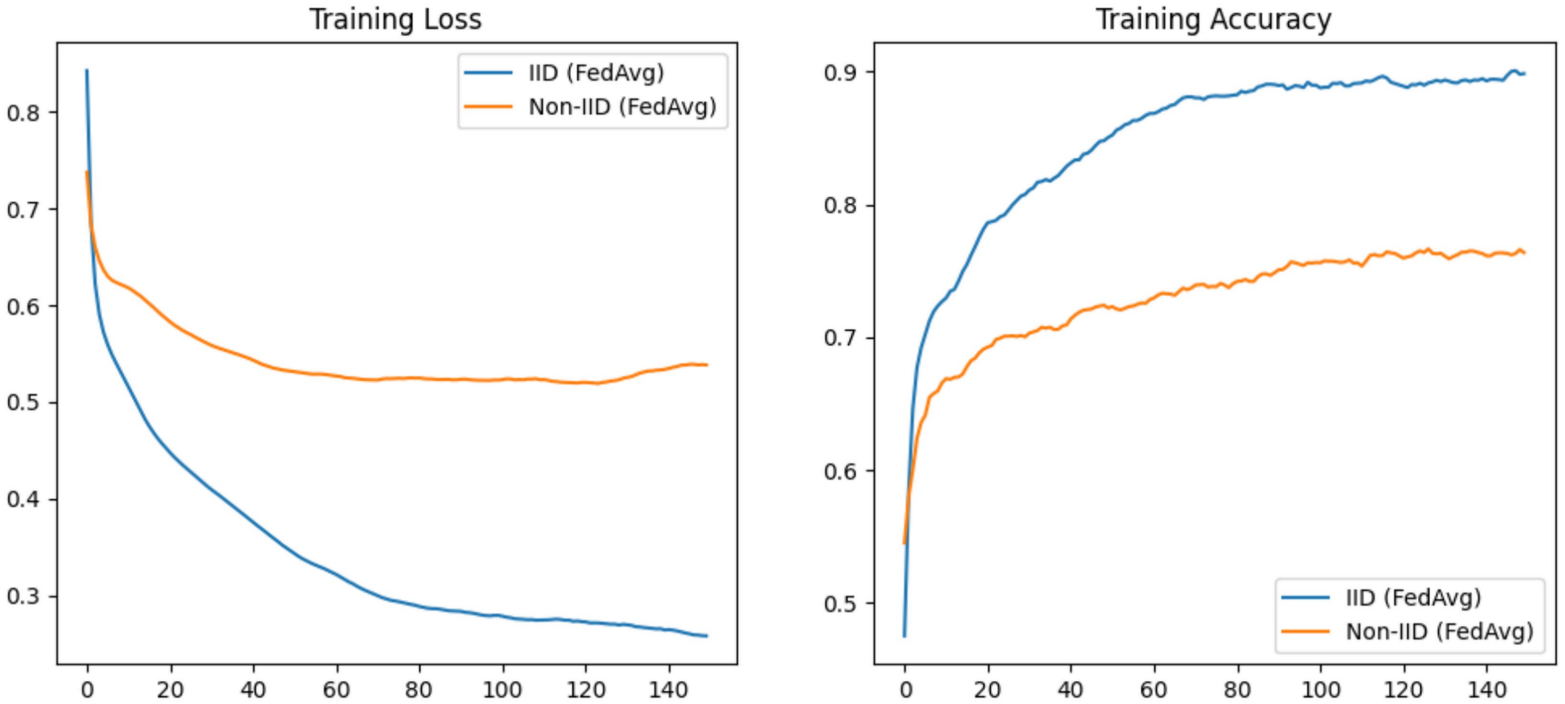
Accuracy and training plots of the baseline case in an IID and non-IID setup using FedAvg with training conducted for 150 rounds.

In an attempt to improve the accuracy in the predictive maintenance performance, we evaluated the performance of a baseline federated learning model using Federated averaging (FedAvg) (McMahan et al., 2017; Li et al., 2019). We applied FedAvg across five (5) clients (with later simulations using ten (10) to twenty (20) clients). The dataset was partitioned into IID and non-IID configurations, with training conducted over 150 rounds and five local epochs per client. As expected and illustrated in Figure 5, the IID case exhibited a sharp decrease in loss and a corresponding improvement in accuracy, while the non-IID case showed significantly slower decrease in loss and struggled to achieve comparable performance. The following section provides a theoretical treatment of the FedAvg algorithm to contextualize these observations.

Federated Averaging (FedAvg) is an algorithm for decentralized training in federated learning. Let *w* represent the global model parameters. At iteration *t*, a selected group of clients denoted by $S_{t}$ performs *E* local stochastic gradient descent (SGD) updates on the model denoted in equation (1):


$$w_{i}^{t+1}=w_{t}-\eta \nabla F_{i}(w_{t})$$ (1)

Where η is the learning rate and $F_{i}(w)$ is the local objective function. The server then aggregates the updated models, as denoted in equation (2), by averaging, adjusted according to the sample size contributed by each client:


$$w^{t+1}=\sum_{i\in S_{t}}\frac{n_{i}}{n_{S_{t}}}\;w_{i}^{t+1}$$ (2)

This process is repeated iteratively to converge to a global model.

(c) Data heterogeneity, technical improvements over baseline FL methodology, experiment and discussion

Federated learning often encounters variation in data distributions among clients, commonly referred to as non-IID data, which presents unique challenges for model convergence and generalization. These non-IID behaviors inherent in the data can be modeled through several mechanisms to better reflect real-world scenarios. Two commonly used approaches are Dirichle—distribution based partitioning introducing label skewness and feature distribution skewness (Li et al., 2022; Mang et al., 2023):

1 Dirichlet-based partitioning (label skew)

To replicate practical scenarios involving uneven data distributions among clients in a federated learning environment, a commonly adopted strategy involves Dirichlet distribution-based partitioning, which can be designed to induce label distribution skew across clients. For illustration, let there be *K* classes and *N* clients. For each class *k* ∊ {1, …, *K*}, a probability vector ( $\pi_{1k},\pi_{2k}m\ldots ,\pi_{\mathrm{Nk}}$ ) is drawn from a Dirichlet distribution Dir(*α*), where*α* > 0 is the concentration parameter controlling the degree of data heterogeneity. The sampled vector determines the proportion of samples from class *k* assigned to each client *i* ∊ {1, …, *N*}. A smaller value of *α* results in a more skewed distribution, with individual clients receiving data predominantly from a limited subset of classes, thereby mimicking non-IID scenarios. Conversely, a larger *α* leads to a more uniform distribution of classes across clients, approximating an IID setting. This approach enables controlled experimentation of varying degrees of data heterogeneity with federated learning simulations.

2 Feature distribution skew

In feature skewness, clients possess data drawn from different feature distributions, even if the label distributions remain similar. To illustrate, for a client *i,* data samples ( $x_{i},y_{i}$ ) are drawn from a client-specific joint distribution ${℘}_{i}(x,y)$ , where the marginal feature distribution ${℘}_{i}(x)$ varies across clients, even if ${℘}_{i}(y|x)$ (the conditional label distribution) remains aligned. Feature skewness can arise due to differences in sensor types, demographic variability, or context, leading to a domain shift between clients. This type of heterogeneity challenges models to generalize across variations in feature spaces.

Other common forms of heterogeneity in federated learning include quantity skew, where clients possess differing amounts of data ( $n_{i}$ varies significantly across clients), and concept drift, where the conditional distribution ${℘}_{i}(y|x)$ differs across clients, reflecting variations in labeling practices or evolving tasks over time. Together with label and feature skew, these variations model the key challenges of decentralized learning environments.

In the context of power system sensor data for predictive maintenance, label skew and feature skew offer more realistic and appropriate means of emulating non-IIDness than quantity skew. Label skew reflects the fact that different substations or assets often experience distinct types of faults or operational states, leading to naturally imbalanced event labels across monitoring sites. Feature skew captures variations in sensor readings arising from differences in equipment models, environmental conditions, operational loads, and maintenance histories. In contrast, quantity skew, where clients have differing amounts of data but similar distributions, is much less probable and inherently fails to represent the critical heterogeneities that directly impact model generalization and failure prediction in real-world electrical infrastructure. Therefore, for data heterogeneity, modeling label and feature skew better aligns with the operational diversity inherent in power system maintenance environments.

Now that a theoretical framework has been established in terms of partitioning the data, based on labels and features, the next section of the discussion shall focus on federated learning solutions that are specifically crafted to deal with data heterogeneity. These solutions are:

1 Adaptive aggregation strategy using FedAvgM (federated averaging with momentum)

At its core, Federated Averaging with momentum (Sun et al., 2024) modifies the basic FedAvg algorithm by incorporating a momentum term into the server-side model aggregation, with the goal of promoting faster convergence and stabilizing model updates despite variations in local client distributions and training dynamics.

Following the standard setup as seen with FedAvg, let $w_{t}\in {\mathbb{R}}^{d}$ denote the global aggregated model during communication iteration *t*, and let each client *i* perform local updates to obtain $w_{t+1}^{i}$ , with $p_{i}$ as defined before. The server maintains the momentum buffer $m_{t}\in {\mathbb{R}}^{d}$ , initialized as $m_{0}=0$ , and updates it according to equation (3):


$$m_{t+1}=\mu m_{t}+\sum_{i=1}^{N}p_{i}(w_{t+1}^{i}-w_{t})$$ (3)

Where μ∈[0,1) is the momentum coefficient.

Then, the global model is updated using as denoted by equation (4) :


$$w_{t+1}=w_{t}+m_{t+1}$$ (4)

In this formulation $\left(w_{t+1}^{i}-w_{t}\right)$ represents the local model change from client *i*. The server aggregates these changes weighted by $p_{i}$ and applies the momentum smoothing using the μ parameter.

Thus, FedAvg with momentum can be interpreted as applying a form of server-side momentum to the aggregated model updates, promoting stability and faster convergence, especially in settings with heterogeneous data distributions.

**Figure 6:**
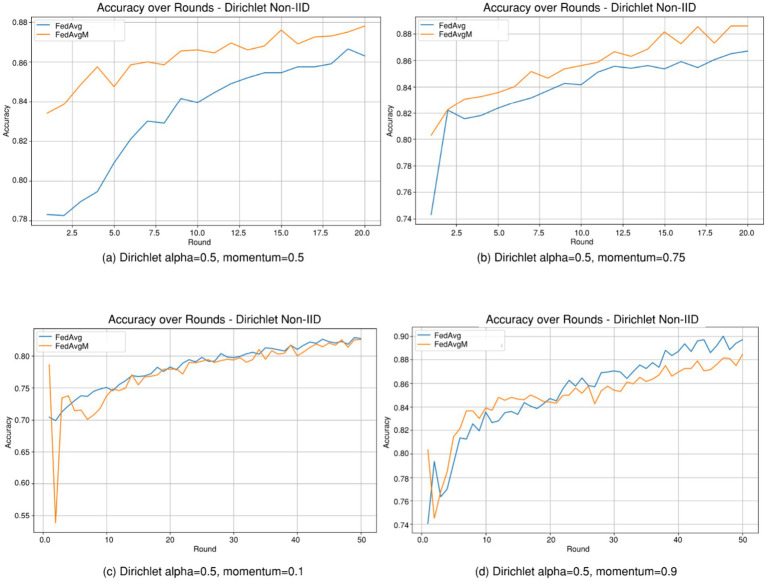
Dirichlet Non-IID performance comparison for FedAvg versus FedAvgM. **(a,b)** FedAvgM with *μ* = 0.5, 0.75, **(c)** FedAvgM with *μ* = 0.1, **(d)** FedAvgM with *μ* = 0.9.

To understand the advantage gained using FedAvgM over FedAvg, simulations are conducted on the *HV Circuit Breaker Maintenance Data* for a balanced Dirichlet partition (*α* = 0.5) with different FedAvgM momentums (*μ* = 0.5 and 0.75, 0.1, and 0.9). From Figure 6 (a and b) one may observe that for a momentum coefficient of *μ* = 0.5 and 0.75, FedAvgM outperforms FedAvg by a decent margin. The momentum parameter, μ, requires careful adjustment, as too low (*μ* = 0.1) of a momentum causes the FedAvgM algorithm to perform similar to FedAvg; as in Figure 6c, while too high of a momentum (*μ* = 0.9) can cause overshooting, oscillations, or instability, especially when the data heterogeneity is strong; see Figure 6d.

2 Modification of local training objective using FedProx

Federated Proximal (FedProx) is an extension of the standard Federated Averaging (FedAvg) algorithm, designed to address challenges arising from system and statistical heterogeneity among clients (Zheng et al., 2024). With FedProx, each client *i* at communication round *t* solves a modified local optimization problem, as denoted through equation (5):


$$\min f_{i}(w)+\frac{\mu }{2}\|w-w_{t}\|2$$ (5)

Where $f_{i}(w)$ denotes the local objective function for client *i*, $w_{t}$ represents the global model parameters at round t, and μ > 0 is a proximal term coefficient controlling the strength of regularization. The additional proximal term $\frac{\mu }{2}\|w-w_{t}\|2$ penalizes deviations from the global model, thereby encouraging local updates to remain close to $w_{t}$ and mitigating issues caused by client drift, especially under non-IID data distributions. After local updates, the server aggregates the updated models (typically via weighted averaging) to form the next global model $w_{t+1}$ . By tuning μ , FedProx provides a flexible mechanism to balance between allowing personalized local updates and maintaining global consistency.

3 Personalization of normalization layers using FedBN

Federated Batch Normalization (FedBN) is another federated learning algorithm designed to mitigate client data heterogeneity (Li et al., 2021) by decoupling the aggregation of batch normalization parameters. Let the model parameters at client *i* be denoted as $\theta_{i}=(\theta_{i}^{\text{shared}},\theta_{i}^{\mathrm{BN}})$ , where $\theta_{i}^{\text{shared}}$ comprises all non-batch-normalization parameters (e.g., convolutional and fully connected layers) and $\theta_{i}^{\mathrm{BN}}\;$ includes the batch normalization parameters, namely the learnable scale and shift parameters ( $\gamma_{i},\beta_{i}$ ) and the running statistics (mean $\mu_{i}$ and variance $\sigma_{i}^{2}).$ Each participating client independently optimizes $\;\theta_{i}^{\text{shared}}\;\text{and}\;\theta_{i}^{\mathrm{BN}}$ using stochastic gradient updates on its local data. Following this local optimization, only the shared parameters $\theta_{i}^{\text{shared}}$ are transmitted to the coordinating server for model integration, as illustrated in equation (6).


$$\theta_{i}^{\text{shared}}\leftarrow \sum_{i=1}^{N}\frac{n_{i}}{n_{\text{total}}}\;\theta_{i}^{\text{shared}}$$ (6)

Where $n_{i}$ is the quantity of local samples at client *i*, and $n_{\text{total}}=\sum_{i=1}^{N}n_{i}$ . The batch normalization parameters $\theta_{i}^{\mathrm{BN}}$ are retained locally, enabling each client to maintain personalized normalization statistics that reflect its own feature distribution. This selective aggregation improves generalization under non-IID client distributions.

**Figure 7:**
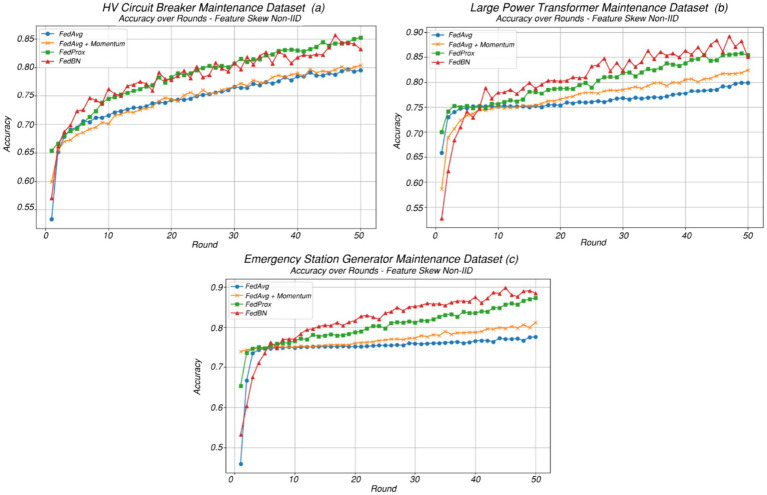
Plot of test set accuracy versus communication rounds for baseline FedAvg versus state-of-the-art FL methods for handling non-IID: FedAvg, FedAvgM, FedProx, and FedBN. **(a)** accuracy over rounds for *HV Circuit Breaker Maintenance Dataset*, **(b)** accuracy over rounds for *Large Power Transformer Maintenance Dataset* **(c)** accuracy over rounds for *Emergency Station Generator Maintenance Dataset*.

Each of the three state-of-the-art approaches for handling non-IIDness—FedAvgM, FedProx, and FedBN, were implemented on the three datasets: *HV Circuit Breaker Maintenance Dataset*, *Large Power Transformer Maintenance Dataset*, and *Emergency Station Generator Maintenance Dataset*, with Figure 7 providing the accuracy over the communication round iterations, with Tables 4, 5 docketing the general parameters/hyperparameters and the F1 scores.

**Table 4:** Selection of parameters and hyperparameters (based on grid search) for the state-of-the-art FL methods (FedAvgM, FedProx, and FedBN) for all three data sets.

Parameters	Values/ Range
Neural network geometry	Dataset geometry/32/16/1
Learning rate	Client optimizer learning rate 0.02 Server optimizer learning rate 0.5–1.0
Activation function	relu ➔ relu ➔ sigmoid
Optimizer	SGD or Adam (SGD selected)
Epochs (client) and communication rounds	10 and 150
Batch size	10
*FedAvgM* ( μ)	0.5 and 0.75
*FedProx* ( μ)	0.1

**Table 5:** F-score results with three major equipment sensor datasets with various data distributions, number of clients, and FL algorithms.

Dataset and partition type	Number of clients	FedAvg	FedAvgM	FedProx	FedBN
*HV Circuit Breaker Maintenance Dataset*—*iid*	10	0.72	0.75	0.80	0.74
	20	0.72	0.72	0.79	0.68
*HV Circuit Breaker Maintenance Dataset—non-iid*	10	0.70	0.69	0.78	0.75
	20	0.69	0.68	0.75	0.72
*Large Power Transformer Maintenance Dataset—iid*	10	0.68	0.73	0.82	0.88
	20	0.68	0.70	0.80	0.86
*Large Power Transformer Maintenance Dataset—non-iid*	10	0.65	0.68	0.72	0.75
	20	0.65	0.66	0.70	0.74
*Emergency Station Generator Maintenance Dataset—iid*	10	0.65	0.66	0.8	0.85
	20	0.65	0.64	0.8	0.82
*Emergency Station Generator Maintenance Dataset—non-iid*	10	0.60	0.65	0.74	0.82
	20	0.60	0.60	0.74	0.80

### 4.1 Inferences and recommendations

The following inferences can be made from Figure 7 and Table 5.

- FedBN (Federated Batch Normalization) decouples batch normalization layers during aggregation, allowing each client to retain local batch statistics (mean and variance). In predictive maintenance, equipment health signatures differ across utilities due to unique operating environments and degradation profiles. FedBN accommodates these local shifts without enforcing global normalization statistics, which would otherwise degrade performance under feature skew.
- FedProx introduces a proximal term that penalizes divergence from the global model, helping stabilize training in the presence of label skew. This constraint helps prevent local models from overfitting their skewed class distributions, which is especially important in maintenance datasets where failure events are rare and unevenly distributed across utilities.
- FedAvgM in contrast yields only marginal gains over FedAvg, as the incorporation of momentum partially accelerates convergence but does not fundamentally address client drift or statistical divergence. In our experiments, FedProx maintained better convergence and generalization on minority failure classes, reducing overfitting observed in vanilla FedAvg under similar conditions.
- The Emergency Station Generator Maintenance Dataset dataset exhibits lower statistical heterogeneity, such as more homogeneous feature distributions, class balance compared to the HV Circuit Breaker Maintenance Dataset converge more consistently with the global objective, allowing the model to achieve its optimal performance within fewer rounds.

The reasoning behind this observation is largely attributed to the fact that unlike high-voltage circuit breakers, which vary significantly in make, interrupting medium (SF₆, vacuum, etc.), age, and operational environment, emergency generators are typically procured as modular backup systems. Substation emergency generators usually conform to similar capacity classes, usage patterns (e.g., periodic testing or standby operation), and maintenance schedules.

- With a larger pool of participating clients, a slight reduction in F-score is observed under non-IID conditions, primarily driven by heightened data variability and fewer samples available per client.

o FedBN and FedProx tend to retain higher F-scores even as client counts grow, due to their mechanisms for reducing drift (e.g., local batch norm stats or proximal regularization).
o FedAvg and FedAvgM may experience sharper F-score degradation under high client counts if data is strongly non-IID.

Based on the inferences made the following recommendations are documented:

- During the initial setup of predictive maintenance programs across utilities, it is essential to ensure that sensor data is collected using standardized units, consistent sampling frequencies, and that the equipment being modeled exhibits comparable operational characteristics.

**Figure 8:**
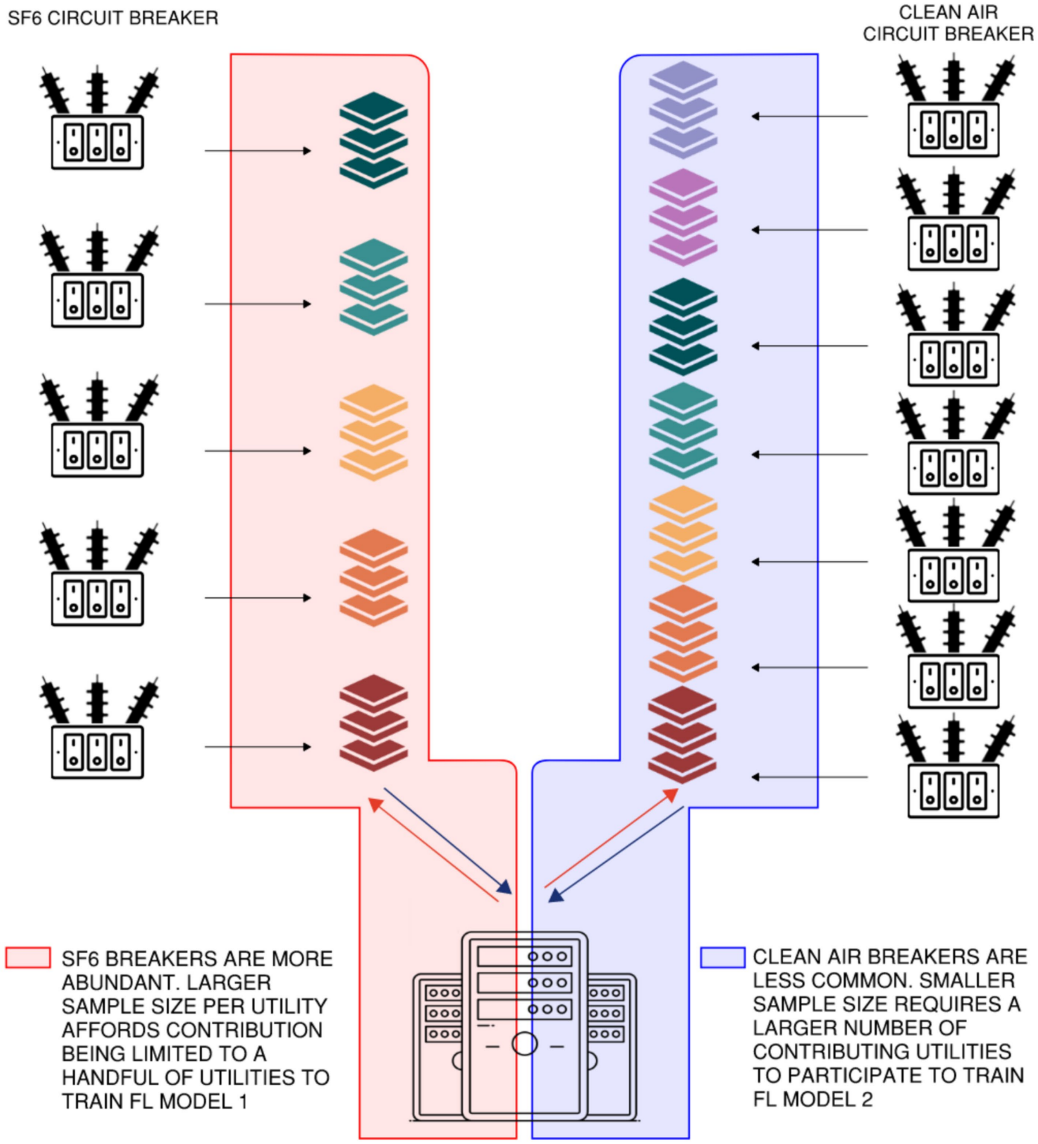
Separation of FL training models for clean air and SF6 breakers to prevent data heterogeneity by design. Participating utilities and central servers maintain model separation.

For example, although both are rated at 138 kV, clean air breakers (Siddiqui et al., 2022) and SF₆ breakers operate based on fundamentally different interruption technologies and produce distinct sensor signatures. As such, these two breaker types should not be grouped within the same federated learning model, as doing so could skew the learning process and degrade model performance. Instead, they should be trained separately, as illustrated in Figure 8 to preserve the integrity of learned patterns. Moreover, due to the relatively limited deployment of clean air breakers across individual utilities, a larger pool of utilities may need to be federated to assemble a representative training sample for this equipment class.
In contrast, SF₆ breakers with similar voltage ratings, such as 138 kV and 230 kV, tend to exhibit comparable sensor profiles and may be clustered together. Overall, federated learning should be implemented by grouping equipment into homogeneous operational classes, thereby reducing data heterogeneity and enhancing model accuracy and generalizability.

- For large power transformers, dissolved gas data are obtained from cooling liquid samples taken from the main tank. The concentration profiles of individual dissolved gases vary depending on the type of cooling liquid used, typically mineral oil or synthetic esters. It is important to recognize that data heterogeneity can arise when comparing dissolved gas measurements from mineral oil-based transformers with those containing synthetic esters, due to their distinct chemical decomposition characteristics. Such data heterogeneity should be identified at the onset of the experimental design, and thought should be given to balancing the different oil transformer types within each client set.
- A similar argument can be made about the fact that emergency generator sensor data can vary based on the fuel type, propane versus natural gas or diesel. Data heterogeneity between emergency generators with different fuel types could be addressed by balancing the component samples on a per client basis.
- Adaptive client sampling should be considered; by preferentially selecting clients whose updates align well with the global objective.
- At an implementation level, certain layers or statistics (e.g., BatchNorm in FedBN) should be allowed to remain client-specific while sharing the global backbone, thereby reducing negative transfer from misaligned data.

## 5 Federated learning-based information criterion (FIC)

In federated learning, data heterogeneity, where data distributions vary significantly across clients, is a central challenge that degrades model performance and convergence. To address this, numerous advanced state-of-the-art algorithms have been developed, each introducing distinct strategies to mitigate the effects of non-IID data. Examples include FedProx, which adds a proximal term to the local objective to stabilize updates; FedDANE, which incorporates second-order local updates using gradient corrections; FedBN, which avoids sharing batch normalization layers to accommodate feature shift; SCAFFOLD, which uses control variates to correct client drift; and FedNova, which normalizes updates to account for client variability in computation. Additional methods such as MOON, Ditto, and FedCurv also target various aspects of personalization and regularization under heterogeneity. Given this diverse algorithmic landscape, selecting a federated learning strategy solely based on predictive accuracy can be short-sighted (Li et al., 2020). Accuracy often overlooks crucial trade-offs such as communication cost, model complexity, and the ability to generalize across client populations. To support more balanced and principled model selection, a Federated Information Criterion (FIC) is proposed, which incorporates both traditional model selection principles and federated-specific penalties.

The Federated Information Criterion (FIC) being proposed in this manuscript is an extension of the classical model selection metrics such as AIC and BIC (Chakrabarti and Ghosh, 2011) to the federated learning setting. Traditional criteria like AIC and BIC do not account for distributed training burdens, which are core to FL scenarios. Federated learning introduces additional challenges including communication overhead, data heterogeneity, and decentralized training. To account for these, the proposed FIC integrates penalties that reflect both conventional and federated-specific costs. Model fit is quantified by the sum of local negative log-likelihoods across clients, while model complexity is penalized based on the total number of trainable parameters. Two additional components are included:

- One representing the overall communication overhead across clients and training rounds, and.
- Another penalizing discrepancy between client-specific updates and the aggregated model, arising from uneven data distributions.

The formal expression for the Federated Information Criterion (FIC) is given as in equation (7):


$$\begin{array}{l} \mathrm{FIC}=\sum_{k=1}^{K}\underset{\text{model}\;\mathrm{fit}}{\underset{︸}{L_{k}({\hat{\theta }}_{k})}}+\underset{\text{communication cost}}{\underset{︸}{\lambda_{1}\cdot C}}\\ +\underset{\text{heterogeneity penalty}}{\underset{︸}{\lambda_{2}\cdot H}}+\underset{\text{complexity penalty}}{\underset{︸}{\gamma \cdot p}} \end{array}$$ (7)

In this Equation 7, $L_{k}({\hat{\theta }}_{k})$ denotes the negative log-likelihood for client *k* serving as a proxy for model fit, *C* is the communication cost (e.g., number of rounds × bandwidth × model size), *H* measures model divergence between local parameters ${\hat{\theta }}_{k}$ and the global aggregate ${\hat{\theta }}_{g}$ , and *p* is the number of model parameters. The constants $\lambda_{1},\lambda_{2}$ , and γ govern the trade-offs among statistical fit, communication efficiency, and model simplicity. The weighting strategy of these constants can be based on a target deployment profile, e.g., prioritizing complexity penalty, followed by heterogeneity penalty, and communication cost in the ratio of 2:1:0.5, representative of a constrained or edge-centric federated learning deployment, or conversely emphasizing communication cost the most, followed by heterogeneity, and only lightly penalizing model complexity for Federated Learning over intermittent or low-bandwidth networks. Additionally, because each component has a different scale and unit (e.g., loss is unbounded, model size is integer-valued, communication cost is in bytes), we apply min-max normalization, see Equation 8, across candidate models to ensure that each term contributes comparably to the final FIC:


$$\tilde{x_{i}}=\frac{x_{i}-\min_{j}(x_{j})}{\max_{j}(x_{j})-\min_{j}(x_{j})+\in }$$ (8)

Where $x_{i}$ is the raw value of a component for model i , and ϵ is a small constant to prevent division-by-zero. If certain penalties (e.g., communication cost) dominate by orders of magnitude, log-scaling may optionally be used before normalization.

The FIC proposed here thereby facilitates informed selection among federated algorithms by accounting for statistical, computational, and infrastructural considerations in a unified metric. It should be noted that non-IIDness is often an inherent property of the data in federated learning; for example, patients from different hospitals, sensors from different substations, or users on different devices naturally generate diverse data. The key point, however, is that how well a federated learning algorithm accommodates or adapts to that heterogeneity should influence model selection, which is precisely what the heterogeneity penalty in the Federated Information Criterion (FIC) aims to capture.

Under theoretical setup, the FIC value can range from large negative to large positive numbers, depending on the balance between predictive loss, model complexity, and federated system costs. Models achieving strong predictive performance with minimal complexity and communication overhead tend to have lower (more negative) FIC scores, while overparameterized or resource-intensive models exhibit higher FIC values. Provides a conceptual comparison of the Federated Information Criterion (FIC) across four commonly used federated learning algorithms, illustrating how each balances model fit, communication cost, heterogeneity handling, and complexity. The comparison in Table 6 highlights why relying solely on accuracy can be misleading in heterogeneous settings.

**Table 6:** Comparative assessment of Federated Information Criterion (FIC) components across common federated learning algorithms.

Algorithm	$\underset{\text{model}\;\mathrm{fit}}{\underset{︸}{L_{k}({\hat{\theta }}_{k})a}}$	$\underset{\text{communication cost}}{\underset{︸}{\lambda_{1}\cdot C}}$	$\underset{\text{heterogeneity penelty}}{\underset{︸}{\lambda_{2}\cdot H}}$	$\underset{\text{complexity penalty}}{\underset{︸}{\gamma \cdot p}}$	FIC value (relative rank)
FedAvg	Moderate to poor (under non-IID)	Low	High (no personalization or correction)	Low	High
FedAvgM	Moderate to good (faster convergence)	Moderate	Moderate	Low	Medium
FedProx	Good (handles drift)	Moderate	Low (proximal term stabilizes)	Moderate	Moderate to Low
FedBN	Good (handles feature skew)	Moderate	Very Low (no BN sharing improves personalization)	Moderate (more parameters)	Low

Assuming a classification task across five clients with moderate non-IID data (Dirichlet *𝛼* = 0.3), simulations were performed, and the FIC values were calculated using: average local log-loss per client, 100 communication rounds, model size of 1.2 MB, and Euclidean divergence between local and global weights. Using normalized penalty weights $\lambda_{1}$ = 0.5, $\lambda_{2}$ = 1.0, and γ = 2, the FIC values obtained for the different cases are:

1. FedAvg: Fit = 1.20, Comm = 0.60, Heterogeneity = 1.10, Complexity = 2.0 ⟹ FIC ≈ 4.90
2. FedAvgM: Fit = 1.10, Comm = 0.65, Heterogeneity = 0.85, Complexity = 2.0 ⟹ FIC ≈ 4.60
3. FedProx: Fit = 1.05, Comm = 0.65, Heterogeneity = 0.55, Complexity = 2.0 ⟹ FIC ≈ 4.25
4. FedBN: Fit = 1.00, Comm = 0.65, Heterogeneity = 0.30, Complexity = 2.4 ⟹ FIC ≈ 4.35

Despite FedBN’s and FedProx’s slightly higher model complexity (due to personalized BN layers or due to the need of modification of local training objective), its ability to significantly reduce inter-client divergence results in the lowest overall heterogeneity penalty, allowing it to outperform others on FIC in this setup, with FedProx gaining the most favorable FIC score in this experimental setup.

In summary, the Federated Information Criterion (FIC) is particularly useful when multiple candidate models exhibit similar accuracy but differ significantly in communication overhead or on-device computation, common in edge-deployed FL scenarios. FIC helps systematically reject over-engineered models that offer diminishing returns relative to their operational cost, providing a principled model selection mechanism for FL under resource constraints. Unlike AIC/BIC, FIC explicitly incorporates FL-specific costs, making it more appropriate for real-world deployments involving battery-constrained, bandwidth-limited, or heterogeneous devices; see Table 7 for comparison. Moreover, it offers tunable flexibility: in scenarios where communication is cheap, but compute is expensive (or vice versa), the penalty weights can be adjusted accordingly.

**Table 7:** Contrasting AIC, BIC, and the proposed Federated Information Criterion (FIC).

Criterion	Centralized data	Penalizes parameters	Penalizes computation	Penalizes communication	Suitable for FL
AIC	Yes	✓	✗	✗	✗
BIC	Yes	✓ (stricter)	✗	✗	✗
FIC	✗ (FL setting)	✓	✓	✓	✓

## 6 The role of dirichlet and alternative distributions in data partition

The role of Dirichlet and alternative distributions - Several commonly used platforms for implementing federated learning, including Flower, FedML, and TFF (TensorFlow Federated) provide built-in utilities to partition datasets using the Dirichlet distribution due to its ease of implementation and tunability. However, these implementations are often heuristic in nature and lack analytical treatment or comparative justification of the Dirichlet distribution’s (Lin 2016) advantages over other distribution-based partitioning strategies, such as Zipf or Beta (Zhu et al., 2018; McDonald and Xu, 1995), in capturing real-world heterogeneity. The authors hope that the subsequent sections cover the analytical gap that exists in current literature.

In federated learning, introducing controlled data heterogeneity across clients is crucial for realistically simulating non-IID settings, particularly under label skew. Among various probabilistic approaches, the Dirichlet distribution, Zipf distribution, and Beta distribution are commonly employed to partition data in a statistically meaningful manner. For a classification task with *K* classes, the Dirichlet distribution models a client’s label distribution $p_{i}=(p_{i1},...,p_{\mathrm{ik}})∼\mathrm{Dir}\;(\alpha .1_{k})$ , where *𝛼* > 0 is a tunable concentration parameter. This allows each client to possess a distinct yet probabilistically valid label mix, with the degree of skew inversely related to *𝛼*.

In contrast, the Zipf distribution generates label frequencies, $P(k;s)=\frac{1/k^{s}}{\sum_{n=1}^{K}1/n^{s}}$ capturing power-law behavior where lower-index classes dominate, useful in mimicking real-world data imbalances but offering less control over client-specific label proportions. Zipf generates a global class distribution, P(k;s)∝1ks not per-client label distributions. That is, it determines which labels are common overall, but not how each client’s dataset should be composed. The Beta distribution, *beta* (α, β) on the other hand requires two parameters *𝛼* and β, to model the proportion of a binary label, with different parameter combinations yield different shapes—uniform, skewed, or peaked across clients. The beta distribution approach is limited to binary classification and needs more parameter tuning per client group, making it less scalable for multiclass tasks. While both Zipf and Beta can induce skew, they lack the flexible multi-class partitioning and fine-grained control that the Dirichlet framework offers.

The primary advantage of the Dirichlet distribution lies in its mathematical structure: it defines a distribution over the *K*-dimensional probability simplex, ensuring that all generated label distributions are valid (non-negative and summing to one) and tunable through a single scalar. This makes Dirichlet-based partitioning both practical and theoretically robust for federated learning experiments involving multiclass tasks and heterogeneous client populations.

**Figure 9:**
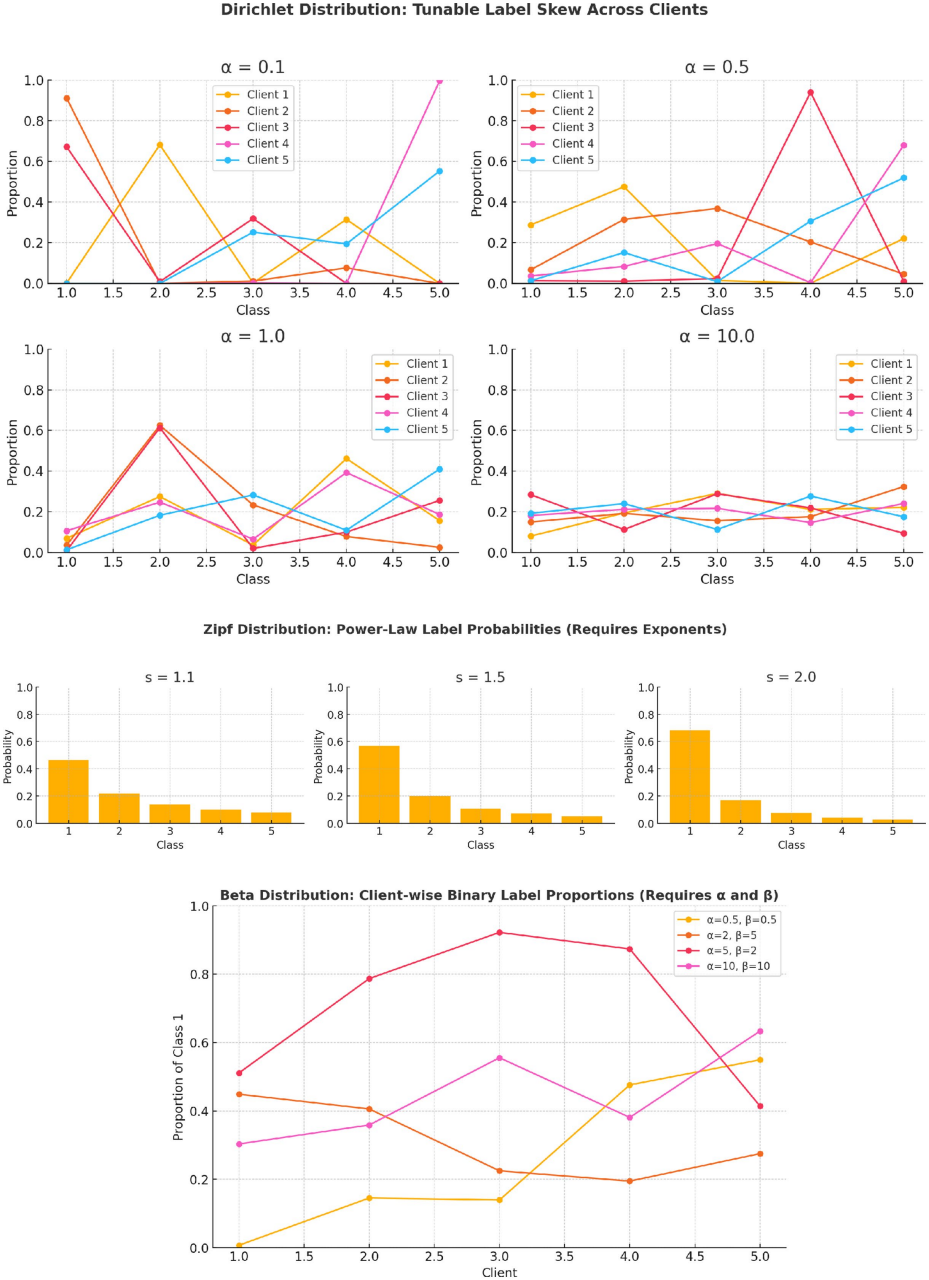
Visual comparison of distribution-based label skew generation methods in federated learning. (Top) Dirichlet distributions with varying concentration parameter, showing smooth control over label heterogeneity across clients. Lower values yield highly skewed distributions, while higher values approach IID. (Middle) Zipf distributions with varying exponent, demonstrating increasing skew toward lower-index classes as increases. Unlike Dirichlet, Zipf does not generate client-specific label mixtures. (Bottom) Beta distributions for binary label proportions across clients with different α, β parameterizations. Beta distribution based partitioning approach enables binary skew modeling but requires two parameters and lacks support for multi-class tasks.

Visualizations in Figure 9 demonstrate the mechanics of how the Dirichlet distribution provides a simple yet powerful mechanism to control label skew across clients in federated learning through a single tunable parameter, while illustrating the complexity of tuning Zipf and Beta distributions for inducing label skew, in contrast to the simplicity of the Dirichlet distribution.

## 7 Summary, challenges, and future work

### 7.1 Manuscript summary

This study presents a novel contribution to the field of predictive maintenance for power systems by demonstrating the applicability of federated learning (FL) frameworks on real-world datasets drawn from high-voltage substation assets. By capturing the nuanced challenges of data heterogeneity, stemming from differences in equipment types, sensor modalities, and data collection protocols across utilities, the work highlights the critical need for algorithmic adaptability in FL deployments.

Among the evaluated methods, Federated Batch Normalization (FedBN) largely outperforms alternatives like FedAvgM and FedProx in handling distributional shifts. However, the observed performance gains are contingent on the nature and granularity of equipment-level data collected at each client node. To support more principled algorithm selection, this study also proposes a Federated Information Criterion (FIC) that balances predictive accuracy with model fit, communication overhead, heterogeneity penalties, and model complexity. These findings emphasize that while tailored FL algorithms offer promise, achieving robust and scalable predictive maintenance solutions requires a deep understanding of the heterogeneity introduced by underlying asset and sensor configurations. This work therefore offers both conceptual foundations and applied direction for implementing federated learning in operational power systems, enabling secure and cooperative asset management at scale.

### 7.2 Implementation challenges

Despite its promise, the practical deployment of federated learning in power system predictive maintenance faces two key implementation challenges. First, inter-utility collaboration is constrained by limited awareness and institutional inertia, with many utilities yet to recognize the full potential of privacy-preserving machine learning for improving asset reliability. Promoting cross-utility partnerships will require sustained engagement, trust-building, and regulatory alignment. Second, sensor calibration inconsistencies across different devices and utilities introduce variability in measurement scales and noise profiles, which can hinder model convergence and reliability. Establishing standardized data preprocessing protocols or sensor harmonization frameworks will be essential to ensure meaningful aggregation across heterogeneous sources.

### 7.3 Future scope of work

This study focused on label skew due to its relevance in modeling class imbalance across clients. Although feature skew was discussed conceptually, it was not experimentally evaluated. This decision was made to maintain model comparability and avoid confounding effects arising from inconsistent feature distributions. Future research may implement controlled feature-skew scenarios, such as covariate shift and conditional divergence, to better evaluate algorithm robustness under realistic federated heterogeneity.

Looking ahead, future work may also be directed towards the exploration of dynamic client clustering based on asset similarity, real-time model adaptation to evolving equipment behavior, and integration of domain-specific priors to further enhance model robustness. Extensions to multi-modal sensor data, secure aggregation techniques, and edge-device optimization will also be critical to operationalizing FL frameworks at scale across diverse utility environments. From a security standpoint, safeguarding FL-based smart grid systems against diverse attack vectors is critical. Byzantine attacks are particularly concerning, where malicious clients inject falsified model updates that can degrade or destabilize the global model. These attacks are notoriously difficult to detect, as they are often indistinguishable from legitimate updates. While robust aggregation techniques have been introduced to counter such threats, further advancements are necessary to ensure resilience under adversarial conditions. Another key vulnerability is the backdoor attack, wherein adversaries embed covert malicious behaviors into the global model while maintaining high accuracy on standard tasks. Mitigating this threat requires strengthening defense strategies such as differential privacy enforcement and anomaly-based detection mechanisms. Although federated learning inherently limits data exposure by keeping raw data local, these indirect leakages remain a concern. Enhancing the use of privacy-preserving techniques, including homomorphic encryption and secure multi-party computation, is essential to bolstering protection against such inference-based threats.

## Data Availability

The data used for this paper can be accessed on the project’s GitHub page: https://github.com/sghosh27/Federated-Learning-for-Critical-Electrical-Infrastructure-Maintenance.
